# Anti-LINGO-1 improved remyelination and neurobehavioral deficit in cuprizone-induced demyelination

**DOI:** 10.22038/ijbms.2021.53531.12043

**Published:** 2021-07

**Authors:** Khadijeh Moradbeygi, Mohsen Parviz, Hossein Rezaeizadeh, Arman Zargaran, Mohammad Ali Sahraian, Shima Mehrabadi, Marjan Nikbakhtzadeh, Elham Zahedi

**Affiliations:** 1 Department of Physiology, School of Medicine, Tehran University of Medical Sciences, Tehran, Iran, Department of Nursing, Abadan Faculty of Medical Sciences, Abadan, Iran; 2 Department of Physiology, School of Medicine, Tehran University of Medical Sciences, Tehran, Iran; 3 Department of Traditional Medicine, School of Persian Medicine, Tehran University of Medical Sciences, Tehran, Iran.; 4 Department of Traditional Pharmacy, School of Traditional Medicine, Tehran University of Medical Sciences, Tehran, Iran; 5 Department of Neurology, Neuroscience Institute, MS Research Center, Tehran University of Medical Sciences, Tehran, Iran

**Keywords:** Brain-derived neurotrophic – factor, Cuprizone, Multiple sclerosis, Myelin basic protein, Nogo receptor 1, Remyelination

## Abstract

**Objective(s):**

Central nervous system demyelination is the main feature of multiple sclerosis (MS). The most important unmet need in MS is use of treatments that delay the progression of the disease. Leucine-rich repeat and Immunoglobulin-like domain containing NOGO receptor-interacting protein 1(LINGO-1) have been known as inhibitors of oligodendrocyte differentiation and myelination.

**Materials and Methods:**

We investigated LINGO-1 antibody effects on remyelination and neurobehavioral deficit using cuprizone-induced demyelination. Animals were randomly divided into three groups (n = 10): (1) Control group; received the regular diet, (2) CPZ group; normal saline was injected intraperitoneally, and (3) Treatment group; LINGO-1 antibody (10 mg/kg) was injected IP once every six days for 3 weeks. We assessed the level of myelin basic protein (MBP), neurofilament heavy chain (NF200), and Brain-derived neuroprotective factor (BDNF) in the corpus callosum (CC) by immunostaining against MBP, NF200, and BDNF.

**Results:**

We found decreased levels of MBP, NF200, and BDNF in demyelinated CC, and anti-LINGO-1 treatment improved demyelinated structures. Furthermore, motor impairment was measured by Open-field (OFT) and Balance beam tests. In the treatment group, motor impairment was significantly improved.

**Conclusion:**

These results provide evidence that LINGO-1 antibody can improve remyelination and neurobehavioral deficit.

## Introduction

Multiple Sclerosis (MS) is a debilitating (1), demyelinating, and neurodegenerative disease of the CNS (2, 3) that is more prevalent in young women than men with diagnosis peaking at about age 40 (4-6). It is known as the most common cause of non-traumatic neurological disability in the United States and Europe (7). According to the National MS Society, MS affects 400,000 Americans and more than 2 million people worldwide (8). MS occurs following damage to the myelin sheath due to autoimmune inflammatory response (9, 10). In patients with MS, neurodegeneration occurs over time as a result of incomplete demyelination and autoimmune reactions (4). 

The cause of MS is unknown and pathological (11). There is no prescriptive medication to help the prevention of neurodegeneration in MS. Medications currently approved for MS treatment are immunomodulators, modulating the immune cell behavior (4). As there is an increasing loss in neuronal function during the progressive stages of MS, (12) improvements in myelin regeneration, have emerged as one of the most important goals of delaying, preventing, or reversing progression in preclinical and clinical research (13). One of the most important causes associated with inhibited remyelination in patients with MS is failure or arrest of oligodendroglial differentiation in MS lesions (14, 15). 

Several mechanisms have been considered to block Oligodendrocytes Progenitor Cell (OPC) maturation, including pathways involving Notch-1, Wnt, LINGO-1, hyaluronan, and Retinoid X receptor (RXR). LINGO-1 inhibits maturation of oligodendrocytes (16, 17). According to previous studies, LINGO-1 antibody improved myelin sheath formation and MBP expression *in vitro* (12). Clearly, in LINGO-1 KO mice, the levels of mature oligodendrocytes (OLs) and myelination percentages were increased (18). 

According to the results of several studies, the LINGO-1 antagonist can improve myelin formation of oligodendrocytes (19, 20). Previous studies have shown that LINGO-1 blockage improves remyelination in experimental autoimmune encephalomyelitis (EAE) mice (21, 22) and other models by inducing oligodendrocyte progenitor cell induction *in vitro *(23, 24). CPZ, a copper chelator, is one of the most common animal experimental models for toxic demyelination (25-27) that is used to assess the mechanisms of oligodendrocyte turnover, astrogliosis, and micro-gliosis (28, 29). Given that oligodendrocyte differentiation and myelination process are essential for CNS development, understanding how these processes are regulated can be effective in providing new treatments of demyelinating diseases such as MS. Therefore, there is a need for therapies that prevent myelin destruction or improve CNS remyelination. In most previous studies, LINGO-1 antibody effects on remyelination in EAE mouse models of MS have been investigated (21,30,31) and the only study using the cuprizone model to evaluate the LINGO-1 antibody effects on remyelination was performed by Sun *et al.* in 2016 (22). They showed that the CPZ model leads to mild impairment in spatial learning while causing significant demyelination in the hippocampus. After treatment with anti-LINGO-1, the learning ability slightly improved MBP expression (22). So, in the present study, we investigate LINGO-1 antibody effects on remyelination in the corpus callosum and behavioral changes in the context of demyelinating conditions. 

## Materials and Methods

***Animals***

Thirty male adult C57BL/6 mice weighing 20–30 g (8 weeks) were purchased from Baqiyatallah University of Medical Sciences (Tehran, Iran). Animals were kept under standard conditions (3 mice/cage,12 hr light/dark cycle; 21–23 °C) and had free access to water and food. All experiment procedures were performed according to the standards established by Tehran University of Medical Sciences ethical committee (approved ID: IR.TUMS.MEDICINE.REC.1399.130). 

***LINGO-1 treatment***

The LINGO-1 antibody (orb469946, Biorbyte, United States) began in the third week, because significant demyelination is detectable after 3 weeks in the cuprizone-fed mice (28). Based on previous experimental studies, LINGO-1 antibody (10 mg/kg ) was intraperitoneally injected once every six days (22). 

***Experimental design***

The mice were randomly assigned into 3 groups (n=10): Control, MS+ Cuprizone-fed group, and Treatment group. Before experiments, animals were kept in plastic cages under controlled environmental conditions, then MS groups were fed cuprizone (bis-cyclohexanone oxaldihydrazone; Sigma-Aldrich) mixed into animals’ normal diet for six weeks to induce toxic-demyelination. 

**Figure 1:**
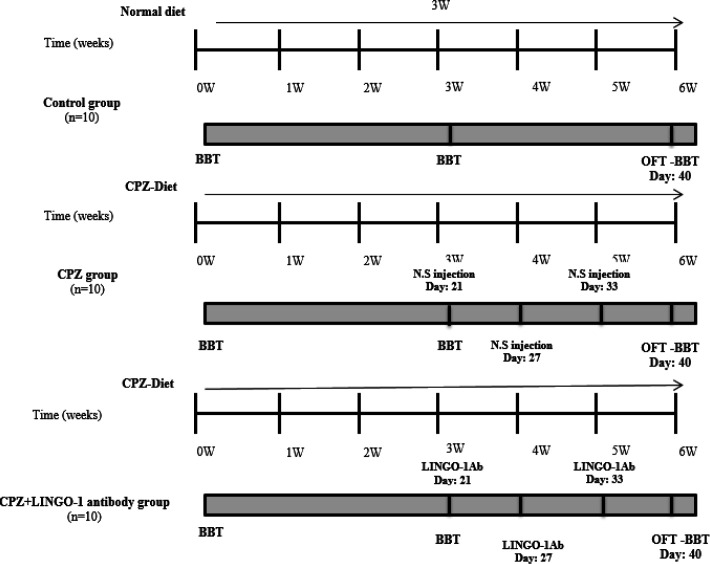
Schematic representation of the experimental protocols. The control group was fed normal chow. The cuprizone group was fed a 0.2% cuprizone (CPZ) diet for 6 weeks without return to normal chow. Behavioral assessments were conducted in the beginning, at three-weeks, and the end of the experimental period, and brain tissue was collected for IHF assessment

BBT: balance beam test; OFT: open field test, Ab: antibody; N.S: normal saline

In order to investigate LINGO-1 antibody effects on remyelination and neurobehavioral deficit, LINGO-1 antibody (10 mg/kg) was injected (IP) in the third week (once, for 6 days). The animals, in the positive control group (CPZ-fed without treatment), were administered 0.9% NaCl. The cuprizone diet, continued until the end of the experimental period (Figure 1).

***Cardiac perfusion***

For transcardial perfusion, mice were anesthetized by ketamine (80 mg/kg) and xylazine (10 mg/kg), IP at the end of the experimental period, and perfusion of phosphate-buffered saline (5 ml) and 4% neutral formalin was used to fix brain tissues. Brains were removed and placed in formalin (10%). Then, kept in 10% formaldehyde solution at ambient temperature for 24 hr. 

***Processing and sectioning of brain tissues***

The brain was separated from the skull, postfixed in 4% paraformaldehyde, and cryoprotected by overnight incubation in sucrose 30%, at 4 °C for 1 to 2 days. Brain tissues were microdissected, dehydrated, and embedded in paraffin. Five-micrometer tissue sections were made in the corpus callosum using a Cryostat (Histoline, Italy). The sections were stored at -20 °C until final staining.

***Immunofluorescence for detection of MBP, NF200, and BDNF***

The brain sections were washed 3x with PBS. Sections were incubated in blocking solution (consisted of 0.1% Triton X-100 (Sigma-Aldrich), 1% bovine serum albumin, and 5% normal serum in PBS) for 1 hr, and then incubated overnight at 4 °C with primary antibody: anti-MBP (anti-rabbit polyclonal; 1:300; Abcam), anti BDNF (anti-rabbit monoclonal; 1:500; Abcam) and anti-NF200 (anti-rabbit polyclonal; 1:10000; Abcam). The day after, sections were rinsed with ice-cold PBS-tween and then incubated with the secondary fluorescence-labeled antibody for 1 hr at room temperature with goat Alexa Fluor 488 F (ab) anti-rabbit IgG as the secondary antibody at 1:1000 dilution. The sections were coverslipped and then images were captured from the stained frozen sections using a Labomed TCS 400 fluorescent microscope for consequent analysis. Table 1 provides the details of the primary and secondary antibodies used in this experiment. 

Brain images were quantified using the ImageJ software. Quantification was performed in three sections per mouse. The sections from all mice were immunostained at the same time. The semi-quantification of the intensities was evaluated using ImageJ software (version 1.50e, U.S. National Institute of Health). The fluorescence signal profile was normalized by the background intensity. Areas related to the corpus callosum in one-half of the brain were analyzed for myelination intensity, BDNF, and NF200 intensity (%). 

***Open field (OF) test***

The open field test (OFT) is the most common method used to assess behavioral research in animal studies which evaluates the quantity and quality of exploratory behavior and activity of animals. For this purpose, the mice are placed into the center of an apparatus and multiple behavioral parameters such as distance moved, time spent moving and rearing are recorded over a set time (5 min). Some results (defecation, center time, and hyperactivity) may indicate some aspects of emotional problems such as anxiety in mice (32). The open-field test was done on the 42nd day post CPZ- feeding. 

***Balance beam test (BBT)***

Measurement of motor coordination and balance can be useful to detect motor deficits caused by CNS lesions in rodents. This test can be more sensitive than Rotarod for some types of motor coordination deficits. At first, the animals are trained for 5 days before the initial assessment just to make sure that the mice’s behavior reflects motor coordination accurately during testing. Then, the balance state of the animals can be evaluated through the delay time to cross the beam. The purpose of this test was to assess the mice’s ability to maintain balance on a wooden beam (1 meter long) which was elevated 30 cm above the ground (33). 

A score of 2 was applied for navigating the beam with full weight support and keeping balance. If plantar placing of the paw was only partly possible, the animal scored 1.5. A score of 1 was given if the animal could cross the entire beam but without plantar placing of the hind paws. When only half of the beam could be crossed, a score of 0.5 was assigned, and for complete inability, a score of 0 was applied (34, 35). Beam test was performed before induction of demyelination with CPZ diet (baseline evaluation) and then at the end of the experimental period. 

***Statistical analysis***

Statistical analysis was performed using GraphPad Prism 4 and SPSS 18 software packages. One-way analysis of variance ANOVA followed by Tukey’s post-test was used to compare the results of the study groups. Results were expressed as mean ± SEM. Differences were considered statistically significant when at least a 95% confidence level was achieved (*P*<0.05). For all graphs, statistical significance is indicated by *P<0.05, **P<0.01, ***P<0.001, and ****P<0.0001. 

## Results

***LINGO-1 antibody improves MBP levels in corpus callosum of CPZ-fed mice***

**Figure 2:**
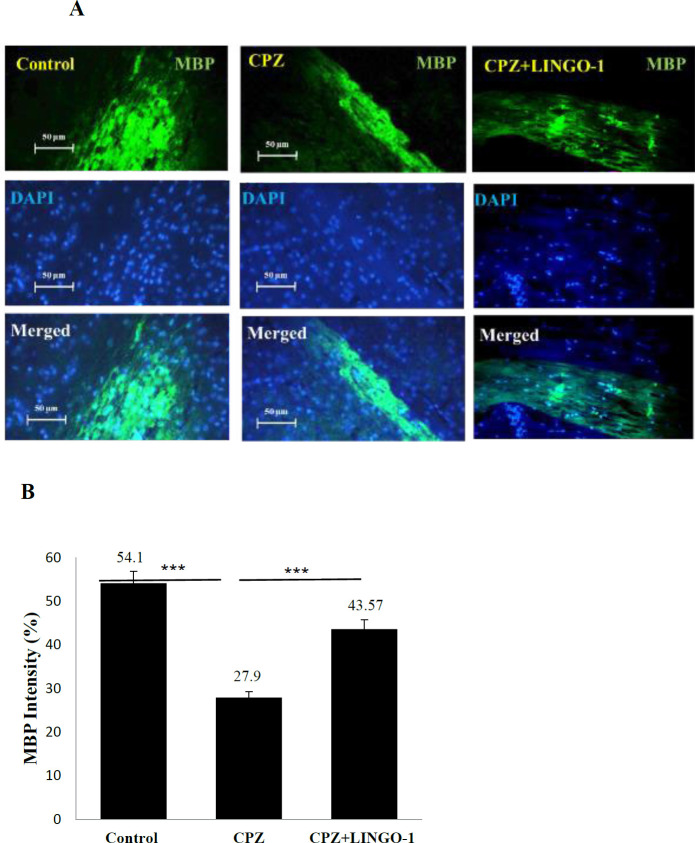
Determining the myelination levels by staining against myelin basic protein (MBP) as a myelin marker in the corpus callosum (CC). (A) Immunofluorescence staining against MBP to evaluate the myelination level in CC. (B) Quantitative analysis of the MBP-stained sections shows the protective effect of the LINGO-1 antibody. Data were shown as Mean±SEM. MBP was increased in the treatment group in comparison with the control group (*P*<0.001). Also, there was a significant decrease in MBP in the CPZ+fed group in comparison with the control group (*P*<0.001). One-way ANOVA was used for statistical analysis followed by Tukey's *post-hoc* test

MBP is a protein that is responsible for the multi-lamellar structure of myelin (35). MBP is expressed by OLs as the main protein in the myelin structure (13). Therefore, measuring MBP levels provides a more accurate method to detect changes in myelination. The LINGO-1 antibody effects on the improvement of demyelination were examined by MBP immunofluorescence staining (Figures 2A, B). We found that the level of MBP in corpus callosum of mice fed cuprizone decreased significantly but increased in the treatment group after treatment with LINGO 1 antibody (Figure 2A, *P*<0.001). Furthermore, the results showed that the level of MBP in the CPZ+ LINGO-1 antibody group was significantly higher than that in the CPZ group (Figure 2A, *P*<0.001). 

***LINGO-1 antibody promotes NF200 level in the corpus callosum of CPZ-fed mice***

**Figure 3:**
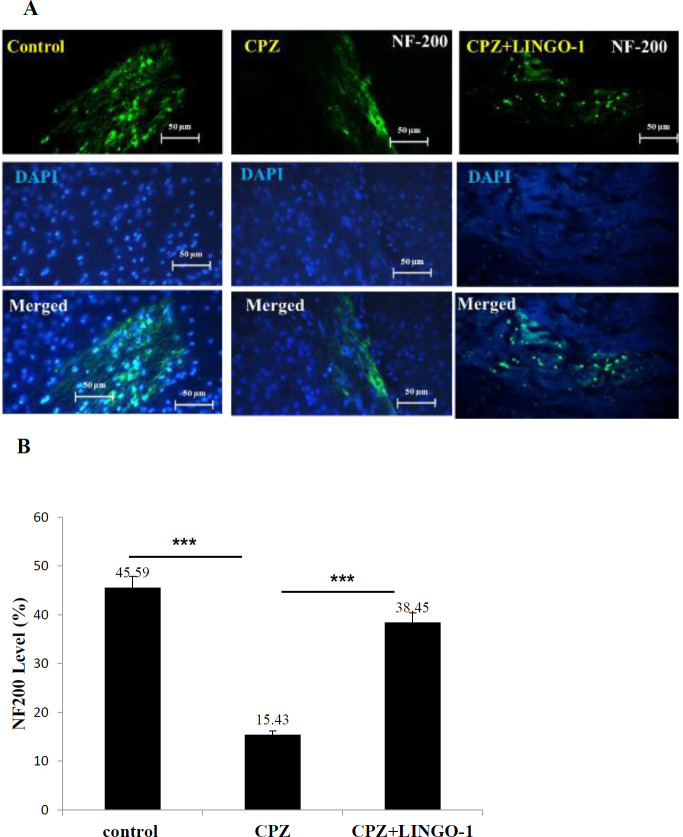
Effect of LINGO-1 antibody on NF200 level in the corpus callosum (CC). (A) The level of NF200 in CC (B) Quantitative analysis of NF200 in sections of CC compared between the three groups. Data were shown as Mean±SEM. NF200 level was increased in the treatment group (*P*<0.001) and decreased in the CPZ+fed group in comparison with the control group (*P*<0.001). One-way ANOVA was used as statistical analysis followed by Tukey's *post-hoc* test. Data presented as mean ± SEM

To evaluate the number of axons in the corpus callosum, we used immunofluorescence staining for NF200. The neuro-filaments are some of the major components of the neuronal cytoskeleton involved in providing structural support for the axon and essential for axonal network formation (22). In corpus callosum, NF200 levels were lower in Coprizone-fed mice than in control mice (Figures 3A, B. *P*<0.001), and after treatment with LINGO- 1 antibody were increased significantly (Figure 3, *P*<0.001). These results showed that LINGO-1 administration might be effective in improvement of demyelination disease. 

***LINGO-1 administration significantly increased BDNF level in the corpus callosum***

**Figure 4:**
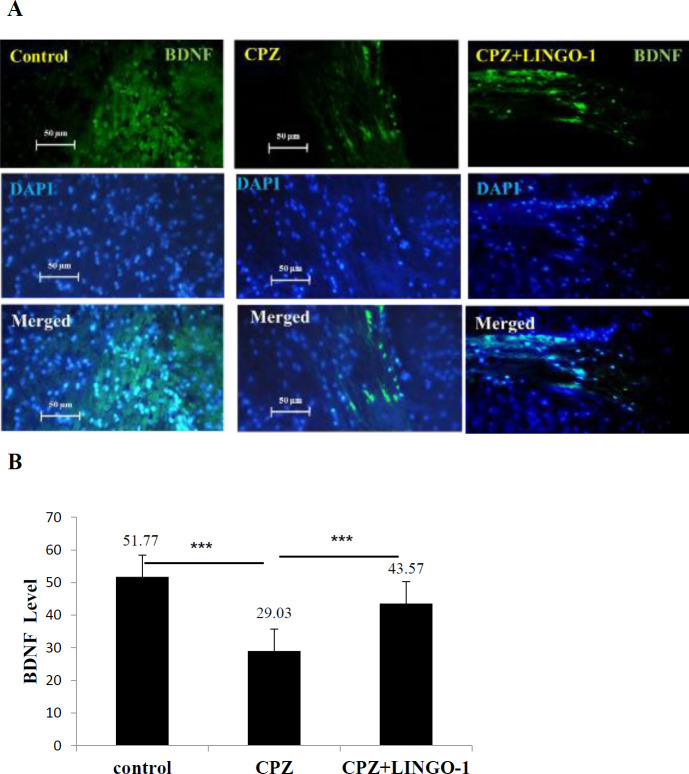
Effect of Anti-LINGO 1 on BDNF. IHF staining against BDNF (A) was performed to investigate the effect of LINGO-1 antibody on BDNF in CC for control, cuprizone, and cuprizone+LINGO-1 groups. (B) Quantitative analysis of BDNF in sections. Data were shown as Mean±SEM. BDNF level was increased in the treatment group (*P*<0.001) and decreased in the CPZ+fed group in comparison with the control group (*P*<0.001). One-way ANOVA was used for statistical analysis followed by Tukey's *post-hoc* test. Data presented as mean±SEM

BDNF concentrations were evaluated in the brain tissue of experimental groups to compare the LINGO-1 antibody effects on BDNF levels in the corpus callosum. The results showed a significant decrease in the brain BDNF levels of mice in the CPZ group (Figures 4A, B. *P*<0.001) compared with the control group. The results showed that the level of BDNF in the treatment group was significantly higher than in the CPZ group (Figures 4A, B. *P*<0.001). 

***LINGO-1 antibody administration improved behavioral test***

**Figure 5:**
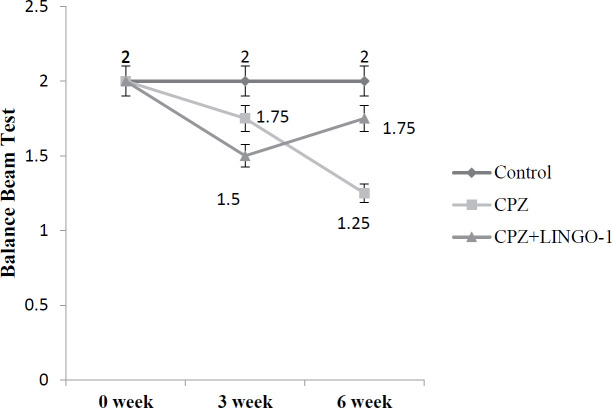
Schematic representation of mice's balance beam test. Values represent mean ±SEM

The mice’s behavior was assessed using OFT at the end of the study period. But the balance beam test was performed at the beginning and the 6th week of the experimental period. LINGO-1 antibody treatment started during the third week of the study. Experimental animals were assessed in OFT to evaluate their level of motor activity. The results showed that LINGO-1 antagonist administration increased the number of squares crossed (Table 2, *P*<0.001) and the number of rears (Table 2, *P*<0.01) in OFT. In the CPZ group, the number of squares crossed and the number of rears decreased compared with that in control mice fed normal chow (Table 2). Using the balance beam test, we investigated the ability of mice to navigate across a 1-m-long beam. The median scores for all groups in the baseline and six weeks were presented in Figure 5. Furthermore, in the balance beam test score assessment, the cuprizone group of mice had worse performance compared with the control group. The latency to reach the platform in the cuprizone group was longer compared with the control group in the 6th week of the experimental period (Figure 5). And after treatment, the latency in the cuprizone mice was similar to that in control mice at the end of the study (Figure 5). 

**Table 1:** List of antibodies used in this study

Supplier	Dilution	catalog	Specificity	Host	Marker
**Abcam**	1:300	ab40390	Mouse, Rat	Rabbit	**MBP**
**Abcam**	1:10000	ab8135	Mouse, Rat	Rabbit	**NF200**
**Abcam**	1: 500	Ab108319	Mouse, Rat	Rabbit	**BDNF**

Ctrl: animals that received normal food and water; CPZ: animals that received normal water and cuprizone food; CPZ+LINGO-1: animals that received cuprizone food and LINGO-1 antibody. ***P<0.001

Balance beam test was performed once before LINGO-1 antibody injection (BL), once at three weeks after CPZ-induced demyelination, and at the end of the experimental period. The balance beam performance of 30 animals is presented as the Mean±SEM. The peak deficits were observed at three weeks and the major recovery took place within six weeks. Correlation between treatment and cuprizone groups in 3 weeks (*P*<0.05) and the end of the study (*P*<0.001) was significant

**Table 2:** Effect of LINGO-1 antibody treatment on some open field parameters recorded from adult C57BL/6 mice. Values represent mean±SEM. In the present work, mice of the treatment group showed a significant increase in the number of squares crossed and rears with respect to other experimental groups. It could be suggested that systematic administration of LINGO-1 antibody increased the locomotor activity as well as the exploration of mice

**Parameters measured**	**Control**	**CPZ-fed**	**CPZ+LINGO-1 **	*P*-value
Number of squares crossed	161 ± 6	92 ± 5	131± 8	*P*<0/001
Number of rears	24 ± 2	17± 6	19 ± 9	*P*<0/05
Number of grooming	11.5 ± 2.5	10 ± 1.5	11 ± 2	*P*>0/05
Number of fecal boli	4 ± 0.5	5 ± 1	4 ± 1	*P*>0/05
Number of urinations	2 ± 0.2	1± 0.5	2 ± 1	*P*>0/05

## Discussion

MS is a debilitating CNS autoimmune disease consisting of CNS-directed inflammation, demyelination, and axonal degeneration (36). In this study, the LINGO-1 antibody administration effect on remyelination and behavioral change in the Cuprizone model of demyelination was investigated. Feeding with a diet containing 0.2% cuprizone caused demyelination and behavioral change. Treatment with LINGO-1 antibody could improve balance beam test scores and OFT results. Furthermore, the intake of 0.2% cuprizone for six weeks led to significant demyelination in the brains of cuprizone-fed mice (37). CPZ feeding induced marked reduction in myelin and MBP staining and decreased levels of NF200 and BDNF in the corpus callosum.

In MS, demyelination occurs after damage to the myelin sheath of neurons due to impaired immune system function. The immune system mainly attacks MBP, a protein that stabilizes and maintains the structure of the myelin sheath around the axon (38). According to previous research, MBP as one of the main proteins in myelin structure plays an essential role in myelin sheath compression and maturation (37, 39). Therefore, any abnormality in the myelin sheath is involved in the deficiency of neuronal function (40). 

Due to the tight relationship between myelin and axonal transport, any dysfunction of myelin can contribute to various types of axonal pathology (22). MBP is associated with the myelin membrane and shows high sensitivity to the concentration of metal ions. Cuprizone administration can destabilize the MBP-membrane binding, reduce myelin density, and degrade myelin quality (35). Consistent with our results, in the previous studies, significant demyelination was detected after 3 weeks in CPZ-fed mice (22, 41). Combined with previous research (22), our results showed that LINGO-1 antibody treatment increased MBP levels compared with the CPZ group. 

Neurofilaments are the intermediate filaments of neurons and some of the main components of the neuronal cytoskeleton (42) which are involved in the provision of the structural support of axons (43). Less organized axo-skeleton may be an early sign of axonal pathology in animal studies (44). There is a report that shows the effect of LINGO-1 antibody treatment on kinesin light chain (KLC) expression in the Parahippocampal cortex (PHC) of the EAE mice (28). In this study, we found that the LINGO-1 antibody could increase NF200 levels in the corpus callosum in the cuprizone-induced demyelination. Axon damage is the hallmark of numerous neurological disorders including MS (45). The proinflammatory cytokines secreted by the activated microglia/macrophage may cause axonal damage. Current immunomodulatory therapies are effective in reducing the recurrence of MS but do not repair disabilities. Therefore, there is a need for neuroprotective therapies to improve disabilities, possibly through increasing remyelination (46). Therefore, our results demonstrate LINGO-1 antibody improved NF200 in the corpus callosum when administrated intra-peritoneal injection for 3 weeks after cuprizone-induced demyelination. According to our results, BDNF levels were significantly decreased in the corpus callosum of the CPZ groups. Previous studies have defined BDNF as a trophic factor that is effective in increasing MBP and MAG expression after demyelination (47). However, despite the fact that glial cells increase BDNF expression following injury, the demyelinating lesion itself causes a decrease in BDNF levels in brain tissue (48). BDNF has a protective role in CNS cells by modulating the release of pro-inflammatory cytokines (49). 

Mohammadi-Rad *et al.* demonstrated that there were interactions between inflammatory and neurotrophic factors that could play an important role in MS symptoms (50). Combined with previous research, our data showed that the use of the LINGO-1 antibody could increase BDNF levels in brain tissue. We also assessed the effect of the LINGO-1 antibody on behavioral change. 

Motor neurobehavioral deficits evaluated using the BBB and OFT after 6 weeks of cuprizone feeding. In OFT, the number of line crosses and the frequency of rearing are usually used as measures of locomotor activity. Thus, the high frequency of these behaviors means increased motor activity (32, 51). In the present study, mice of the treatment group showed a significant increase in the number of rears compared with the CPZ+MS group. It could be suggested that the LINGO-1 antibody can increase locomotor activity.

With the balance beam test, we investigated the ability of mice to navigate across a 1-m-long beam. According to our data, there was a significant difference between experimental groups at the end of the experimental period (*P*<0.001). Furthermore, the mice in the cuprizone-fed group showed a lower score in the beam test at week 6, and the marks improvement was seen at the end of the study in the treatment group. 

Therefore, it can be concluded that LINGO-1 injection can improve the beam test score in the CPZ model of demyelination. The beam’s ability to walk indicates the descending movement control of the vestibulospinal tract. The previous study has indicated persistent deficits in balance beam test at early stages of EAE (33) that provides a valuable test for the evaluation of balance, tail function, and descending motor control in an animal model of experimental autoimmune encephalomyelitis. Therefore, we can show that the effects of the LINGO-1 antibody on myelination, probably affect the beam test score in treatment groups. 

## Conclusion

In our study, we found that the use of the LINGO-1 antibody improved remyelination and behavioral changes in the CPZ model of demyelination in mice. The results showed that systemic administration of LINGO-1 antibody improved animal activities maybe through remyelination enhancement. In addition, the increased levels of MBP, NF200, and BDNF were seen in the corpus callosum after the three-week LINGO-1 antibody treatment. Taken together, our study has shown that LINGO-1 antibody may be effective in remyelination and neurobehavioral defects in MS. 
